# Leisure-Time Physical Activity, Time Spent Sitting and Risk of Non-alcoholic Fatty Liver Disease: A Cross-Sectional Study in Puglia

**DOI:** 10.1007/s11606-024-08804-9

**Published:** 2024-05-28

**Authors:** Isabella Franco, Antonella Bianco, Caterina Bonfiglio, Ritanna Curci, Angelo Campanella, Alberto Rubén Osella

**Affiliations:** 1 National Institute of Gastroenterology—IRCCS “S. de Bellis”, Castellana Grotte, Italy; 2 Estadìstica y Bioestadìstica Escuela de Nutriciòn, Facultad de Ciencias Médicas, Universidad Nacional de Córdoba, Córdoba, Argentina

**Keywords:** leisure-time physical activity, time spent sitting, NAFLD

## Abstract

**Background:**

Non-alcoholic fatty liver disease (NAFLD) is the most common chronic liver disease in the world. The increasingly sedentary lifestyle in recent years may have accelerated the development of NAFLD, independent of the level of physical activity.

**Objective:**

The purpose of this cross-sectional study was to determine the association between leisure-time physical activity (LTPA) and time spent sitting (TSS) and the likelihood of developing NAFLD in a sample of men and women aged 18–64 years, from southern Italy.

**Design:**

The study is based on two cohort studies, a randomized clinical trial and an observational cost-benefit study.

**Participants:**

A total of 1269 participants (51.5% women) drawn from 3992 eligible subjects were enrolled in this study.

**Exposures:**

Leisure-time physical activity (LTPA) and time spent sitting (TSS) were assessed using the Italian long form of the International Physical Activity Questionnaire (IPAQ-LF), designed for administration to adults aged 18 to 65 years.

**Main Measures:**

The association of exposures with the probability of belonging to a certain NAFLD degree of severity.

**Key Results:**

The probability of having mild, moderate, and severe NAFLD tends to decrease with increasing LTPA and decreasing TSS levels. We selected a combination of participants aged 50 years and older stratified by gender. Men had a statistically significant difference in the probability of developing moderate NAFLD if they spent 70 h per week sitting and had low LTPA, while among women there was a statistically significant difference in the probability of developing mild or moderate NAFLD if they had moderate LPTA and spent 35–70 h/week sitting.

**Conclusions:**

The study thus showed that the amount of LTPA and the amount of TSS are associated with development and progression of NAFLD, but this relationship is not a linear one—especially in women aged ≥ 50 years old.

## INTRODUCTION

Non-alcoholic fatty liver disease (NAFLD) is the most common chronic liver disease in the world^1^ and encompasses a broad spectrum of liver diseases, ranging from simple steatosis to non-alcoholic steatohepatitis.^2,3^ In recent years, time spent sitting has increased significantly, leading to a rising prevalence of obesity and NAFLD. This association has significant public health implications^4^ on the management of the disease. In the absence of sufficient drug treatment, lifestyle modifications are effective during the treatment of NAFLD: diet and physical activity are factors that play a key role in the pathogenesis and progression of NAFLD.^5,6^ Regular practice of physical activity promotes calorie consumption, whereas low levels of physical activity can worsen the condition of NAFLD.^7^ The protective effect of physical activity on the incidence and mortality of several chronic diseases, including cardiovascular disease, diabetes, stroke, and several types of cancer, is well known.^8^ Recently, the deleterious effects of sedentary behavior, independently of leisure-time physical activity, have received much attention. In fact, several epidemiological studies have suggested a positive association between sedentary behavior and obesity, diabetes, insulin resistance, metabolic syndrome, cardiovascular disease, cancer, and mortality other than that associated with little physical activity.^9–14^ This association, which was more pronounced in subjects with high levels of physical activity (moderate to vigorous), indicates, however, that regular physical activity does not fully protect against risk when associated with prolonged sedentary behaviour.^15^ Therefore, we might hypothesize that prolonged sedentary behavior, regardless of the level of physical activity, even in leisure time, may play a potential role in the development and progression of NAFLD. The purpose of this cross-sectional study was to explore the likelihood of developing NAFLD by associating leisure-time physical activity with time spent sitting in a sample of men and women aged 18 to 64 years, from southern Italy.

## MATERIALS AND METHODS

### Participants and Study Design

This was a cross-sectional study conducted at the National Institute of Gastroenterology “S. de Bellis” (Castellana Grotte, Bari, Italy). The study was based on four studies whose details have been published elsewhere, two being cohort studies, one a randomized clinical trial and one a cost-benefit observational study. We chose to assemble the four studies to account for the geographic differences that characterized the two cohorts, and the study design that provided for different inclusion criteria. In this way, we hypothesized that the heterogeneity of the participants could produce more robust results.

Briefly, the MICOL study (Italian Multicenter Colelithiasis Study) is an ongoing prospective cohort study assembled in 1985 based on a random sample of the Castellana Grotte (Apulian Region, Italy) population aged ≥ 30 years old. The participants were followed up in 1992–1993, 2005–2006, and 2017–2020; the last follow-up was stopped due to COVID-19 restrictions. The main objective of the study was to estimate the prevalence of cholelithiasis and its risk factors but also to probe other liver diseases. The study retrospectively showed a high prevalence of hepatitis C infection and NAFLD.^16^ In this study, we considered only the 2017–2020 follow-up.

NUTRIHEP (Nutrition and Hepatology) is an ongoing prospective cohort study assembled in 2005–2006 based on a random sample of the Putignano (Apulian Region, Italy) population aged ≥ 18 years old. The aim of this survey was to estimate the prevalence of several liver diseases as well as to find out population dietary patterns. The study showed a low prevalence of hepatitis B and C infection and a high prevalence of NAFLD.^17^ The first follow-up was carried out between April 2015 and May 2019.

The NUTRIATT study was a randomized clinical trial (www.https://clinicaltrials.gov, registration number CT02347696), conducted from March 2015 to December 2016. The study enrolled participants aged > 29 to < 60 years old, randomly assigned to six different treatments consisting of a diet, two different physical activity programs, and their combination lasting 6 months. All interventions were useful to reduce NAFLD scores but the combination of low glycemic Mediterranean diet (LGIMD) and an aerobic physical activity program was the most efficient combination.^18^ For this study, we took baseline data.

The COST-BENEFIT was an observational study conducted from March 2018 to February 2020. In this study, participants aged 18–65 years old, suffering from hypertension, dyslipidemia, or type II diabetes or insulin resistance, were recruited. Participants were asked to follow a diet and a personalized physical activity program lasting 1 year. The study was stopped because of the restrictions of COVID-19. The aim of this study was to probe the benefits of a LGIMD and a mixed exercise program on metabolic-associated fatty liver disease. LGIMD and the mixed exercise program significantly improved metabolic-associated fatty liver disease status; in addition, longitudinal body mass index and HOMA-IR measurements were good predictors of the disappearance of diagnostic criteria for metabolic-associated fatty liver disease.^19^ For this study, we took baseline data.

**Figure 1:**
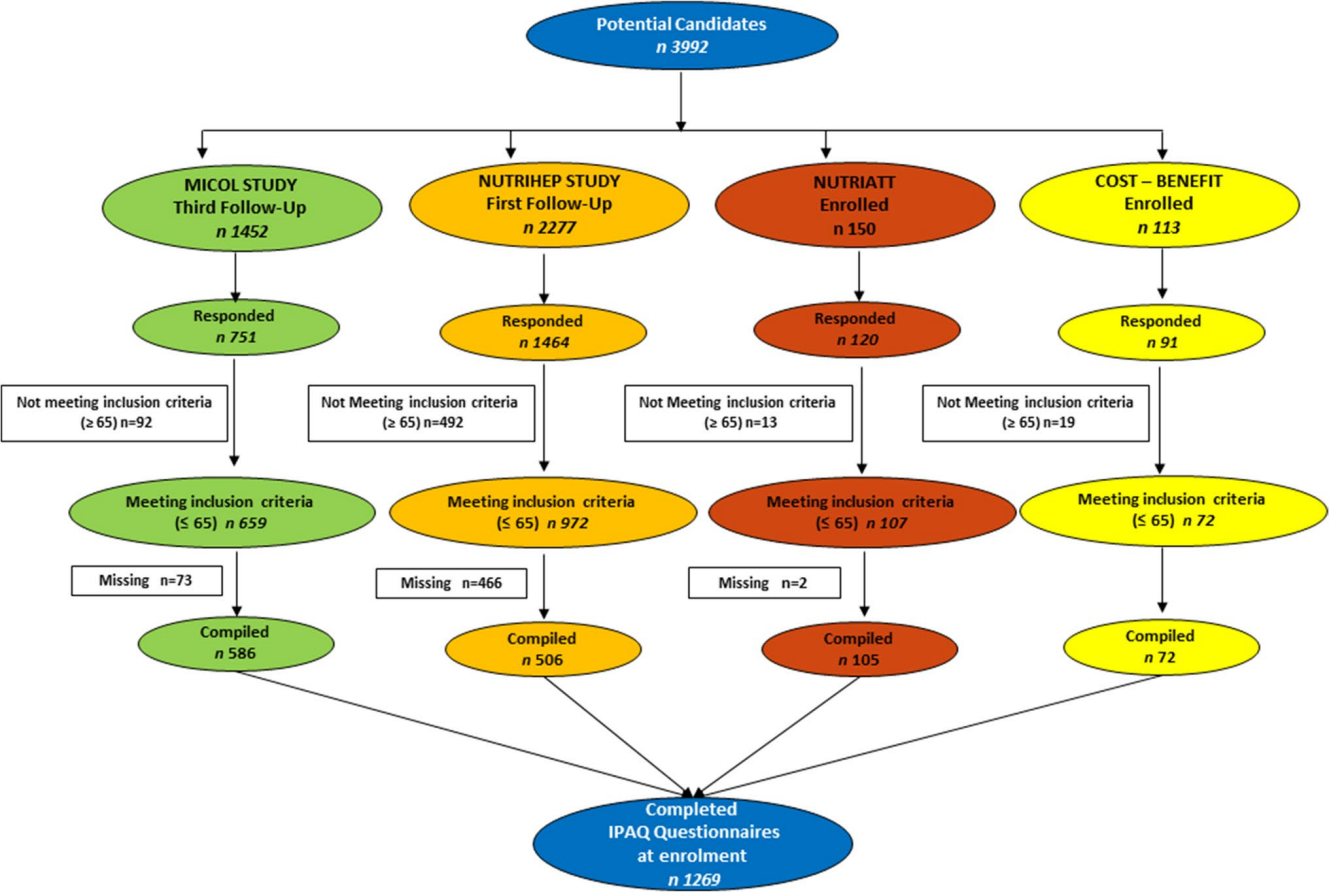
Flowchart of the studies.

The flowchart of the studies, with the number of subjects and other details, is shown in Fig. 1.

All the studies were approved by the Ethics Committee and were conducted in accordance with the Declaration of Helsinki. All subjects signed informed consent.

### Data Collection

In all the studies, trained staff collected data using questionnaires and standardized procedures. Socio-demographic, anthropometric, nutritional, health-related, and lifestyle data were collected. Blood pressure was assessed following international guidelines. Anthropometric measurements (weight, height, waist circumference) were taken in standard manner. Weight and height measurements were taken using SECA instruments (Model 700 and Model 206; 220 cm; SECA, Hamburg, Germany). NAFLD assessment and grading were performed by ultrasound examination (LUS) in the MICOL, NUTRIHEP, and COST-BENEFIT studies and by controlled attenuation parameter in the NUTRIATT study. Fasting blood samples were drawn in the morning and collected in tubes containing ethylenediaminetetraacetic acid (K-EDTA) anticoagulant. Biochemical measurements were performed using standard methods. The following biomarkers were evaluated in all studies: HbA1c, glucose, AST, ALT, GGT, total cholesterol, HDL cholesterol, triglycerides, insulin, HOMA-IR, CRP. The International Physical Activity Questionnaire - Long Form (IPAQ-LF) was self-administered in all studies, but staff provided assistance in reading the questionnaire if needed. All measured values are shown in Table 1.

**Table 1:** Epidemiological and Clinical Characteristics of Patients Enrolled (*n* = 1269), According to the Categories of Leisure-Time Physical Activity (LTPA) and Time Spent Sitting (TSS)

	All sample	LTPA (met min week)			TSS (h/week)		
		< 600	600–3000	> 3000	< 35	35–70	> 70
*N*	1269	444	538	287	742	455	72
Age*	52.47 (7.83)	52.19 (7.66)	52.52 (7.98)	52.81 (7.85)	52.80 (7.68)	51.85 (8.10)	53.03 (7.54)
Age categories (years)^ϯ^							
< 30 years	10 (0.8)	4 (0.9)	5 (0.9)	1 (0.3)	5 (0.7)	4 (0.9)	1 (1.4)
30–40 years	89 (7.0)	27 (6.1)	39 (7.2)	23 (8.0)	50 (6.7)	35 (7.7)	4 (5.6)
41–50 years	326 (25.7)	126 (28.4)	131 (24.3)	69 (24.0)	182 (24.5)	128 (28.1)	16 (22.2)
51–60 years	610 (48.1)	216 (48.6)	260 (48.3)	134 (46.7)	365 (49.2)	206 (45.3)	39 (54.2)
60–64 years	234 (18.4)	71 (16.0)	103 (19.1)	60 (20.9)	140 (18.9)	82 (18.0)	12 (16.7)
Sex^ϯ^							
Female	654 (51.5)	208 (46.8)	302 (56.1)	144 (50.2)	435 (58.6)	198 (43.5)	21 (29.2)
Male	615 (48.5)	236 (53.2)	236 (43.9)	143 (49.8)	307 (41.4)	257 (56.5)	51 (70.8)
NAFLD^ϯ^							
Absent	593 (46.8)	184 (41.7)	257 (47.8)	152 (53.0)	391 (52.7)	187 (41.1)	15 (21.7)
Mild	287 (22.7)	106 (24.0)	116 (21.6)	65 (22.6)	157 (21.2)	112 (24.6)	18 (26.1)
Moderate	245 (19.4)	102 (23.1)	102 (19.0)	41 (14.3)	127 (17.1)	92 (20.2)	26 (37.7)
Severe	141 (11.1)	49 (11.1)	63 (11.7)	29 (10.1)	67 (9.0)	64 (14.1)	10 (14.5)
Marital status^ϯ^							
Single	111 (8.7)	40 (9.0)	48 (8.9)	23 (8.0)	60 (8.1)	45 (9.9)	6 (8.3)
Married or living together	1081 (85.2)	382 (86.0)	455 (84.6)	244 (85.0)	639 (86.1)	378 (83.1)	64 (88.9)
Separated or divorced	54 (4.3)	17 (3.8)	22 (4.1)	15 (5.2)	26 (3.5)	27 (5.9)	1 (1.4)
Widow/er	23 (1.8)	5 (1.1)	13 (2.4)	5 (1.7)	17 (2.3)	5 (1.1)	1 (1.4)
Education^ϯ^							
Primary school	105 (8.3)	31 (7.0)	43 (8.0)	31 (10.8)	81 (10.9)	24 (5.3)	
Secondary school	466 (36.7)	155 (34.9)	186 (34.6)	125 (43.6)	328 (44.2)	126 (27.7)	12 (16.7)
High school	534 (42.1)	187 (42.1)	238 (44.2)	109 (38.0)	270 (36.4)	223 (49.0)	41 (56.9)
Graduate	164 (12.9)	71 (16.0)	71 (13.2)	22 (7.7)	63 (8.5)	82 (18.0)	19 (26.4)
Work^ϯ^							
Managers and professionals	113 (8.9)	33 (7.4)	49 (9.1)	31 (10.8)	51 (6.9)	52 (11.4)	10 (13.9)
Crafts, agricultural and sales workers	614 (48.4)	221 (49.8)	262 (48.7)	131 (45.6)	318 (42.9)	255 (56.0)	41 (56.9)
Elementary occupations	320 (25.2)	107 (24.1)	135 (25.1)	78 (27.2)	204 (27.5)	104 (22.9)	12 (16.7)
Housewife	151 (11.9)	57 (12.8)	64 (11.9)	30 (10.5)	122 (16.4)	26 (5.7)	3 (4.2)
Unemployed	71 (5.6)	26 (5.9)	28 (5.2)	17 (5.9)	47 (6.3)	18 (4.0)	6 (8.3)
SBP (mmHg)*	120.95 (14.37)	121.82 (14.60)	121.22 (14.01)	119.13 (14.58)	120.51 (14.04)	121.29 (14.93)	123.55 (13.99)
DBP (mmHg)*	79.13 (7.96)	79.77 (8.11)	78.96 (7.96)	78.45 (7.68)	78.66 (7.41)	79.68 (8.75)	80.51 (8.05)
BMI (kg/m^2^)*	28.06 (5.20)	28.42 (5.49)	28.13 (5.27)	27.39 (4.51)	27.72 (5.01)	28.27 (5.25)	30.31 (6.26)
WHR* (cm)	91.10 (13.55)	93.20 (14.28)	90.44 (13.39)	89.09 (12.22)	89.38 (12.78)	92.45 (13.95)	100.44 (14.11)
Hip circumference* (cm)	102.22 (10.32)	102.67 (10.91)	102.58 (10.29)	100.87 (9.33)	101.93 (9.99)	102.16 (10.34)	105.67 (12.93)
Daily calorie consumption*	1477.3 (1321.6)	963.60 (1067.7)	1391.2 (1173.5)	2431.8 (1432.7)	1767.4 (1412.0)	1156.7 (1076.2)	500.77 (682.55)
HbA1c (mmol/l)*	0.37 (0.07)	0.37 (0.07)	0.37 (0.07)	0.37 (0.06)	0.37 (0.07)	0.37 (0.07)	0.39 (0.07)
Glucose (mmol/l)*	5.30 (0.98)	5.33 (1.06)	5.30 (1.03)	5.23 (0.70)	5.25 (0.90)	5.33 (1.09)	5.49 (0.98)
AST (μkat/L)*	0.37 (0.12)	0.37 (0.12)	0.37 (0.11)	0.38 (0.14)	0.36 (0.12)	0.38 (0.11)	0.40 (0.14)
ALT (μkat/L)*	0.41 (0.21)	0.43 (0.23)	0.41 (0.21)	0.39 (0.19)	0.39 (0.19)	0.44 (0.23)	0.49 (0.28)
GGT (μkat/L)*	0.34 (0.31)	0.37 (0.29)	0.31 (0.25)	0.33 (0.41)	0.32 (0.33)	0.35 (0.27)	0.38 (0.26)
TC (mmol/l)*	5.14 (0.95)	5.21 (0.94)	5.17 (0.97)	5.00 (0.92)	5.13 (0.96)	5.13 (0.95)	5.38 (0.95)
HDL-C (mmol/l)*	1.30 (0.33)	1.29 (0.31)	1.31 (0.35)	1.30 (0.33)	1.33 (0.34)	1.27 (0.32)	1.22 (0.30)
Triglycerides (mmol/L)*	1.17 (0.75)	1.25 (0.81)	1.16 (0.77)	1.05 (0.61)	1.10 (0.72)	1.24 (0.81)	1.39 (0.72)
Insulin (µUI/mL)*	8.52 (6.33)	9.61 (7.56)	8.30 (5.69)	7.27 (4.99)	7.69 (4.90)	9.41 (7.43)	11.54 (9.54)
HOMA-IR*	2.08 (1.84)	2.37 (2.20)	2.03 (1.76)	1.73 (1.21)	1.86 (1.45)	2.32 (2.16)	2.92 (2.65)
CRP*	0.25 (0.50)	0.26 (0.35)	0.26 (0.68)	0.21 (0.25)	0.25 (0.55)	0.25 (0.43)	0.30 (0.30)

*LTPA*, leisure-time physical activity; *TSS*, time spent sitting; *NAFLD*, non-alcoholic fatty liver disease; *SBP*, systolic blood pressure; *DBP*, diastolic blood pressure; *BMI*, body mass index; *WHR*, waist circumference; *HbA1c*, glycated hemoglobin; *TC*, total cholesterol; *AST*, aspartate aminotransferase; *ALT*, alanine aminotransferase; *GGT*, γ-glutamyltransferase; *TC*, total cholesterol; *HDL-C*, high-density lipoprotein cholesterol; *HOMA-IR*, homeostasis model assessment for insulin resistance; *CRP*, C-reactive protein. Cells reporting subject characteristics contain * Mean±(SD). ^ϯ^Number. (Percentage) Percentages calculated for the column

### Exposure Assessment

The Italian long form of the International Physical Activity Questionnaire (IPAQ-LF)^20^ was used in all these studies, designed for administration to adults aged 18–65 years. IPAQ-LF was self-administered with the assistance of the staff in reading the questionnaire, if needed, to avoid missing and inconsistent values and reduce classification bias. Other than demographic and social characteristics, the questionnaire probes the following domains: household and garden work activities, occupational activity, self-transport, leisure-time physical activity, time spent sitting, and hours of sleep. An additional question was asked about the rate of walking and cycling. Coding and processing were performed following the indications contained in the User Guide of the IPAQ webpage. The information about LTPA was expressed as METxMinutexDay/Week-1 and TSS as h/week.

### Statistical Analysis

Descriptive data analysis was performed using means (SD) and frequencies (%) as appropriate. For descriptive and analytical purposes, exposure assessment was categorized as follows: PA leisure (Met*Min*Week-1) low (< 600), moderate (600–3000), and high (≥ 3000) and time spent sitting (h/week): < 35, 35–70, and > 70.

Subsequently, due to the ordinal scale of the NAFLD degree of severity, a multivariable ordinal logistic model (OLM) was fitted to the data. PA leisure and time spent sitting, the exposure variables, were introduced in the model as principal effects and as modification effects between them. Age (< 50/≥ 50), sex (male vs. female), AST, ALT, daily calorie intake, PCR, and HbA1c were used as adjusting variables.

Results are expressed as odds ratios (ORs) and their corresponding 95% confidence intervals (95% CIs) which estimate the association between the exposures and NAFLD in each of the degrees of severity. The estimated cut-off values for the categories of NAFLD are also expressed as ORs. Using post-estimation tools, the parallel regression assumption was tested by means of the test of Brant (overall test and single variable test), as well as the prediction of probabilities of belonging to each of the exposure categories, and their combination were obtained and then graphically displayed. Moreover, as a result of the OLM, marginal probabilities of some ideal patterns of covariates were estimated and the differences among them were tested. We chose the combination of age and sex as previous results had indicated that this population reached a maximum body mass index between 50 and 60 years old with a higher prevalence of overweight among men.^21^

Statistical analysis was performed using Stata 18.1 (StataCorp LLC, StataCorp, 4905 Lakeway Drive, College Station, TX 77845, USA). In particular, the estimate command -ologit- and the post-estimation commands -predict-, -Brant-, -mtable-, and -mlincom- were used.^22^

## RESULTS

**Figure 2:**
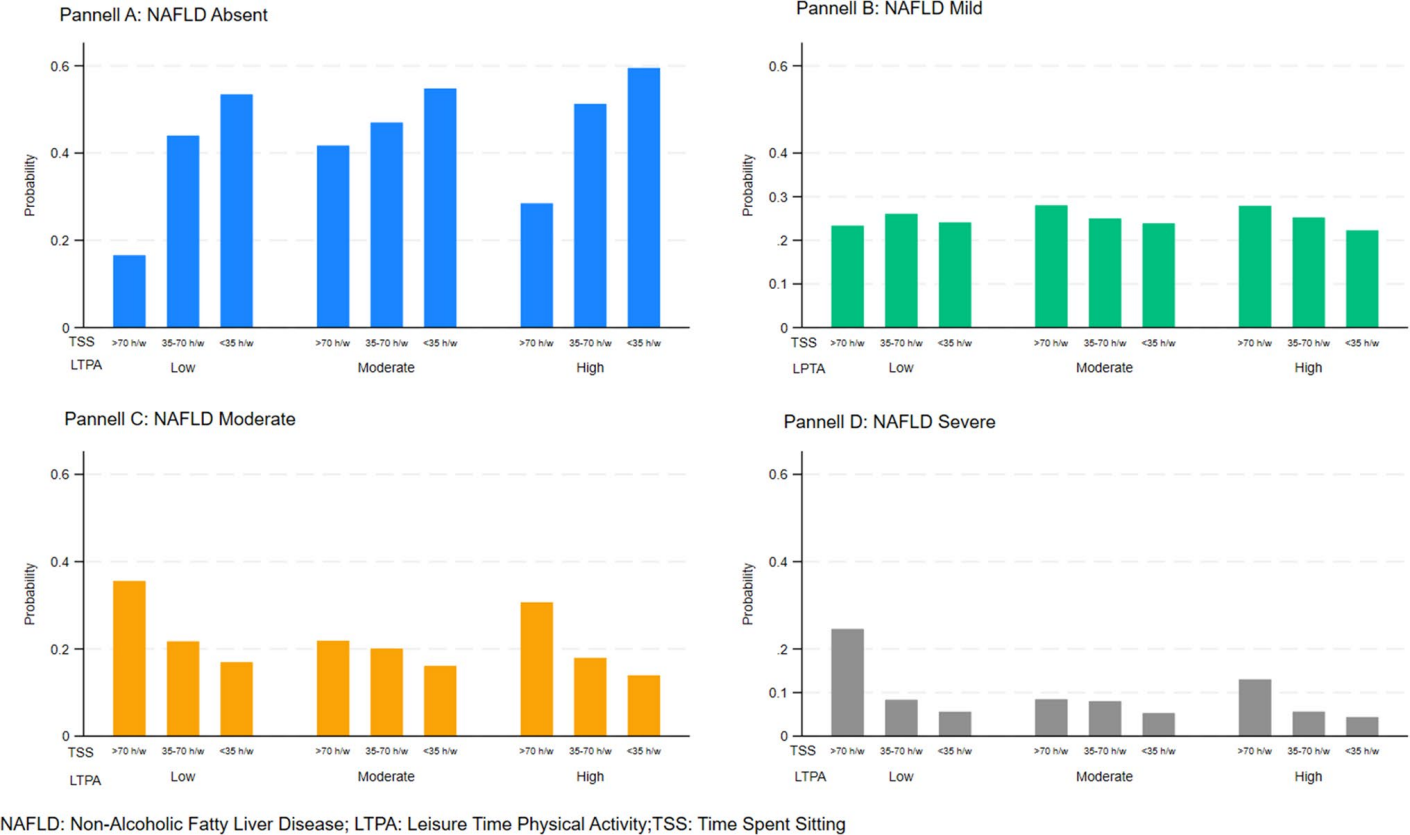
Predicted probabilities of NAFLD by type of exposure and degree of severity. NAFLD, non-alcholic fatty liver disease; LTPA, leisure-time physical activity; TSS, time spent sitting.

A description of the enrolled sample is shown in Table 1. In total, 1269 (51.5% women) participants selected among 3992 eligible subjects were enrolled in this study. Characteristics of eligible and enrolled subjects are shown in Table 1. All enrolled subjects were younger than 66 years old as the instrument used to measure the exposure was validated for the range 18–65 years old. The mean age was 52.47 (7.83) years and about 50% of the participants belonged to the 51–60 years old category; 53.8% had a diagnosis of NAFLD. Most of the participants were married or engaged in stable relationships (85.2%), had secondary school or a higher level of education (91.7%), and were Crafts, Agricultural and Sales Workers (48.4%). Biochemical markers and anthropometrics characteristics of the sample are shown in Table 1. Results from OLM are shown in Table 2. The Brant test of the parallel regression assumption held. There were no principal significant effects of LTPA and TSS. However, there were statistically significant decreased ORs for the modification effect of low LTPA leisure/< 35 h sitting/week (OR 0.30, 95% CI 0.10,0.87), moderate LTPA leisure/< 35 h sitting/week (OR 0.29, 95% CI 0.10, 0.83), high LTPA leisure/35–70 h sitting/week (OR 0.31, 95% CI 0.10,0.94), and high LTPA leisure/< 35 h sitting/week (OR 0.23, 95% CI 0.07,0.68) on NAFLD degree of severity. Post-estimation predicted probabilities are graphically displayed in Fig. 2. As can be seen, the probability of not developing NAFLD is associated with the PA level and sitting time (panel a): the higher the PA level and the lower the time spent sitting, the higher the probability of not developing NAFLD. For mild (panel b), moderate (panel c), and severe (panel d) NAFLD, there is a decreasing trend in the probability of developing NAFLD as the LTPA level increases and time spent sitting decreases. Estimated probabilities of some patterns of covariates and differences in probabilities among them are shown in Table 3. We chose the combination of participants aged 50 years old or older stratified by sex and belonging to the four outcome categories with a low or medium level of PA who spent 70 h/week sitting as compared with those who spent 35–70 h/week sitting. Men had a statistically significant difference in probability of developing moderate NAFLD if they spent 70 h/week sitting and had a low level of PA whereas among women there were statistically significant probability differences of developing mild or moderate NAFLD if they had medium PA and spent 35–70 h/week sitting.

**Table 2:** Ordered Logit Models Between NAFLD Scores According to the Categories of Leisure-Time Physical Activity and Time Spent Sitting

LTPA#TSS	OR*	*p*-value	95% CI
Low # > 70 h/w	1.00		
Low # 35–70 h/w	0.43	0.12	[0.15, 1.24]
Low # < 35 h/w	0.30	**0.03**	[0.10, 0.87]
Moderate # > 70 h/w	0.33	0.11	[0.08, 1.29]
Moderate # 35–70 h/w	0.44	0.13	[0.15, 1.27]
Moderate # < 35 h/w	0.29	**0.02**	[0.10, 0.83]
High # > 70 h/w	0.72	0.72	[0.12, 4.23]
High # 35–70 h/w	0.31	**0.04**	[0.10, 0.94]
High # < 35 h/w	0.23	**0.01**	[0.07, 0.68]

Value highlighted in bold indicate statistical significance (*pv* < 0.005)

^*^Age (< 50 vs. ≥ 50 years old), sex, daily calorie intake, AST, ALT, CRP, and HbA1c-adjusted estimates. *LTPA*, leisure-time physical activity; *TSS*, time spent sitting; *OR*, odds ratio; *CI*, confidence interval; *AST*, aspartate aminotransferase; *ALT*, alanine aminotransferase; *CRP*, C-reactive protein; *HbA1c*, glycated hemoglobin

**Table 3:** Covariate Patterns and Probability of Developing NAFLD

	NAFLD degree of severity probability							
	Absent		Mild		Moderate		Severe	
	Pr	*p*-value diference	Pr	*p*-value diference	Pr	*p*-value diference	Pr	*p*-value diference
Men ≥ 50 years old								
Low PA-sitting 70 h/week	0.33		0.32		0.28		0.08	
Low PA-sitting 35–70 h/week	0.32	0.10	0.32	0.15	0.28	0.02	0.08	0.26
Medium PA-sitting 70 h/week	0.22		0.30		0.35		0.12	
Medium PA-sitting 35–70 h/week	0.37	098	0.32	0.92	0.24	0.93	0.06	0.93
Women ≥ 50 years old								
Low PA-sitting 70 h/week	0.45		0.31		0.20		0.05	
Low PA-sitting 35–70 h/week	0.44	0.35	0.31	0.68	0.20	0.81	0.05	0.33
Medium PA-sitting 70 h/week	0.33		0.32		0.27		0.08	
Medium PA-sitting 35–70 h/week	0.50	0.10	0.29	**0.02**	0.17	**0.02**	0.04	0.06

Values highlighted in bold indicate statistical significance (*pv* < 0.05)

*NAFLD*, non-alcoholic fatty liver disease; *Pr*, probability; *PA*, physical activity

## DISCUSSION

There is strong evidence of the benefits of physical activity.^23,24^ The regular practice of PA reduces the risk of premature mortality and is an effective primary and secondary prevention strategy for at least 25 chronic diseases.^25,26^ However, more than a quarter of the adult population worldwide^27,28^ is not sufficiently physically active, and several studies have shown that sedentary behavior and low levels of leisure-time physical activity are associated with negative health outcomes.^4,10,29,30^ Our study showed that subjects who spend less time sitting during leisure time are more likely to remain NAFLD-free regardless of the level of PA and, those who spend more time sitting during leisure time are more likely to develop more severe forms of NAFLD (mild to severe) when this is accompanied especially with low levels of LTPA, particularly among women aged 50 years old or more. The mechanisms by which sedentary behavior contributes to a higher prevalence of NAFLD, regardless of the level of physical activity, are still uncertain. Similar results to ours have been obtained by previous correlation studies that have identified an association between sedentary behavior and metabolic syndrome, regardless of physical activity levels.^31–36^ Indeed, cross-sectional studies have shown that increased sedentary behavior and low physical activity levels are a key problem at population level and consequently could play a potential role in the development of NAFLD, independently of physical activity.^37–40^ The results of our study show that the amount of time spent sitting and the amount of physical activity performed in leisure time are not linearly associated with the occurrence and degree of severity of NAFLD. The greater the sedentary time, the greater the probability of developing more severe forms of NAFLD. As a consequence and, although the inverse relationship between PA and NAFLD is well established in the literature, our results show that these benefits are not sufficient for individuals who spend a lot of time sitting, as sitting continuously during work or leisure can alter some metabolic parameters and increase overall inflammatory processes.^41^ However, it is possible to diminish inflammation by taking short breaks during sitting^42^, to obtain other health benefits.^43^ Indeed, NAFLD is closely related to unhealthy lifestyles and the positive association between sitting time and NAFLD could also be explained as the consequence of a higher calorie intake and/or lower energy expenditure, resulting in the development of obesity or at any rate weight gain, and an elevated risk of NAFLD.^8,43–45^ In addition, the WHO has drawn up recommendations for leisure-time physical activity to reduce the likelihood of NAFLD development and progression, regardless of shared risk factors.^5,46^ We chose two ideal covariate patterns to estimate the probability of getting NAFLD. In previous studies^21^, we showed a sharp increase in the BMI in persons aged 50 years or older, reaching a maximum at 64.8 years old in this population, with a statistically significant effect of sex. As NAFLD is a correlate of obesity, we took these parameters to estimate and test the difference in probability among subjects with different degrees of leisure-time PA and time spent sitting. Globally, the NAFLD prevalence is higher in men although recent reports show a trend to increasing NAFLD among women.^47,48^ In fact, men were likely to develop moderate NAFLD if they had a low level of physical activity and spent 70 h/week sitting; women, on the other hand, were likely to develop mild to moderate NAFLD if they had a moderate level of physical activity and spent 35/70 h/week sitting. In our study, the results also show that the probability of developing NAFLD is different between men and women aged ≥ 50. The higher prevalence in NAFLD among men is reduced or nullified in women after menopause.^49,50^ The mechanisms underlying this changing risk pattern among pre- and postmenopausal women remain incompletely understood. Several mechanisms have been hypothesized, ranging from findings in animal models to studies in human beings. In animal models, it has been shown that dietary factors can synergistically enhance estrogen deficiencies leading to increased hepatic injury.^51^ The loss of protection conferred by estrogens combined with other disorders (abdominal adiposity, metabolic syndrome, microbiota dysregulation, insulin resistance)^52,53^ could be the underlying conditions leading women to have an increased risk of developing NAFLD as well as other metabolic diseases.^54,55^ Moreover, the relative androgen increase during menopause could contribute to the development of NAFLD.^56^ Some methodological issues need to be considered. The strength of this study is the large number of subjects enrolled. Despite the exclusion of subjects due to missing information, we can assume that the number of subjects may still be representative of the local population and selection bias should be minor. Limitations of the study are related to self-reported sedentary behavior data, as participants’ activity levels were measured and described only using physical activity questionnaires. However, IPAQ has shown to be resistant to serious differential misclassification bias and, if present, it tends toward the null hypothesis. It is worthy to note that the Apulian population is extremely homogeneous, then the ethnic diversity of this setting does not matter.

## CONCLUSIONS

Prolonged sedentary behavior has a greater influence on the development and progression of NAFLD, regardless of levels of leisure-time physical activity. In women over the age of 50, this association is probably more evident due to hormonal changes after menopause. However, it is possible to mitigate the deleterious effects of prolonged sedentary behavior by taking short breaks during sessions, and so also to gain benefit from the protective effect of PA.

## Data Availability

All data are available by contacting the corresponding author.
